# Altered frontocingulate activation during aversive interoceptive processing in young adults transitioning to problem stimulant use

**DOI:** 10.3389/fnsys.2013.00089

**Published:** 2013-11-15

**Authors:** Jennifer L. Stewart, Jason M. Parnass, April C. May, Paul W. Davenport, Martin P. Paulus

**Affiliations:** ^1^Department of Psychiatry, University of CaliforniaLa Jolla, CA, USA; ^2^Psychiatry Service, Veterans Affairs San Diego Healthcare SystemSan Diego, CA, USA; ^3^Department of Physiological Sciences, University of FloridaGainesville, FL, USA

**Keywords:** functional magnetic resonance imaging (fMRI), stimulants, decision making, error processing, interoception, breathing load

## Abstract

Problems associated with stimulant use have been linked to frontocingulate, insular, and thalamic dysfunction during decision making and alterations in interoceptive processing. However, little is known about how interoception and decision making interact and contribute to dysfunctions that promote the transition from recreational drug use to abuse or dependence. Here, we investigate brain activation in response to reward, punishment, and uncertainty during an aversive interoceptive challenge in current and former stimulant (cocaine and amphetamine) users using functional magnetic resonance imaging (fMRI). Young adults previously identified as recreational users (*n* = 184) were followed up 3 years later. Of these, 18 individuals progressed to problem stimulant use (PSU), whereas 15 desisted stimulant use (DSU). PSU, DSU, and 14 healthy comparison subjects (CTL) performed a two-choice prediction task at three fixed error rates (20% = reward, 50% = uncertainty, 80% = punishment) during which they anticipated and experienced episodes of inspiratory breathing load. Although groups did not differ in insula activation or subjective breathing load ratings, PSU exhibited lower right inferior frontal gyrus (IFG) and bilateral anterior cingulate (ACC) activation than DSU and CTL during aversive interoceptive processing as well as lower right IFG in response to decision making involving uncertainty. However, PSU exhibited greater bilateral IFG activation than DSU and CTL while making choices within the context of punishing feedback, and both PSU and DSU showed lower thalamic activation during breathing load than CTL. Findings suggest that frontocingulate attenuation, reflecting reduced resources devoted to goal maintenance and action selection in the presence of uncertainty and interoceptive perturbations, may be a biomarker for susceptibility to PSU.

## Introduction

A growing literature suggests that brain regions involved in interoception, such as insular cortex, are dysfunctional in substance abuse and dependence and may be involved in the maintenance, escalation, and/or relapse of drug use (Paulus et al., 2009; Naqvi and Bechara, 2010; Verdejo-Garcia et al., 2012a). More specifically, substance dependence may reflect a discrepancy between an individual's predicted vs. actual internal state known as the bodily prediction error, an imbalance disconnected from accurate valuation of external stimuli (e.g., current and future rewards and punishments) that may in turn influence the degree of future drug-related approach vs. avoidance behavior (Paulus and Stewart, 2014). For example, inadequate insular functioning in drug users may result in persistent but undetected aversive states, which are unable to modulate cognitive control mechanisms implemented by the prefrontal cortex (PFC) during decision making (Paulus et al., 2009; Verdejo-Garcia et al., 2012a).

With respect to models of the interoceptive system, researchers (Damasio, 1996; Bechara, 2005) have postulated that the insular cortex coordinates with other brain regions to process and integrate somatosensory feeling states in order to guide future decisions. It has been argued that the thalamus delivers sensory information first to the posterior insula and then to the anterior insula, resulting in bodily feeling states that are registered by the anterior cingulate cortex (ACC), which initiates motivated action to regain internal homeostasis and minimize bodily prediction error (Craig, 2003; Paulus et al., 2009). Neuroimaging research supports the role of thalamic, insular, and genual/subgenual ACC function during probing of the interoceptive system (Critchley, 2004; Critchley et al., 2004; Pollatos et al., 2007; Paulus et al., 2012; Zaki et al., 2012). Individuals with substance dependence may have inadequate function in this relay system in response to positive and/or negative body signals to engage in adaptive approach or avoidance behaviors (Paulus and Stewart, 2014). In particular, the interaction between compromised interoception and cognitive control systems involving regions of the PFC may lead to suboptimal decision making (making poor choices such as using drugs despite anticipating and facing negative outcomes). This hypothesis is supported by recent neuroimaging research showing that amphetamine dependent individuals exhibit lower insula, thalamus, ACC, and PFC activation than healthy comparison subjects while making choices in a simple decision making task and concurrently experiencing pleasant interoceptive stimuli via soft bristle brush (May et al., 2013).

Some studies have shown that stress alters frontocingulate, thalamic, and insular regions in stimulant dependent individuals, leading to heightened craving and relapse (Sinha et al., 2006, 2007; Sinha, 2007). For instance, cocaine-dependent patients exhibit lower ACC activation than healthy comparison subjects during exposure to non-drug related stressful imagery (Sinha et al., 2005), whereas the presence of stress (mild shock to the wrist) is associated with greater thalamus and ACC activation than the absence of stress within the context of drug cues in a small sample of cocaine dependent men (Duncan et al., 2007). Moreover, a recent study examining gender differences in responses to neutral, stress, and cocaine imagery scripts indicates that although cocaine dependent men and women both exhibit greater thalamus activation during stress provocation than healthy subjects, cocaine dependent women show greater insula, ACC, and PFC activation than healthy women in response to stress (Potenza et al., 2012). Taken together, these findings support the assertion that in stimulant dependent individuals, aversive states are associated with heightened neural processing as well as urges to engage in drug-related approach behavior. However, additional research is warranted to determine whether aversive interoceptive states influence valuation of external stimuli when stimulant users are making decisions in the face of both positive and negative consequences.

Individuals with stimulant dependence demonstrate neural and behavioral dysfunction within the context of decision making. For instance, amphetamine dependent patients exhibit impaired behavioral performance (altered win-stay patterns of responding) and attenuated insular and PFC activation when making decisions during varying outcome contexts involving reward, uncertainty, and punishment (Paulus et al., 2002b, 2003, 2005). Moreover, amphetamine and/or cocaine dependent individuals show insular, ACC, PFC, and/or thalamic attenuations in paradigms involving reward evaluation (Goldstein et al., 2007; Monterosso et al., 2007; Hoffman et al., 2008), moral judgments (Verdejo-Garcia et al., 2012b), selective attention and working memory (Bolla et al., 2003; Kubler et al., 2005; Tomasi et al., 2007a,b; Clark et al., 2012), response conflict (Salo et al., 2009; Nestor et al., 2011) and behavioral inhibition (Kaufman et al., 2003; Connolly et al., 2012).

On the whole, neuroimaging studies of decision making indicate that the insular cortex, thalamus, PFC, and ACC subserve many functions that may be impaired in stimulant dependence. However, studies involving the intersection of decision making and interoception are still warranted to address the role of neural and behavioral function in stimulant users, particularly in response to aversive bodily signals that may drive stimulant use. Moreover, it is still unclear whether alterations in frontocingulate, insular, and thalamic regions are: (1) markers of the susceptibility to experiment with stimulants more generally; (2) present in the early stages of problem stimulant use (PSU; e.g., recent onset abuse and/or dependence); or (3) indicators of chronic stimulant use only. If neural mechanisms involved in decision making and interoception are impaired in the transition to problem use as well as in chronic use, neuroimaging can be utilized as an early detection tool to motivate more intensive interventions for high risk individuals.

To address these questions, the present study utilized functional magnetic resonance imaging (fMRI) to study decision making during an aversive interoceptive manipulation in a sample of young adults with varying levels of stimulant use. A two-choice prediction task with fluctuating error rates was employed to examine decision making in response to rewarding, uncertain, and punishing outcomes. In addition, an inspiratory breathing load shown to activate insula and PFC during decision making was used as an aversive interoceptive manipulation (Paulus et al., 2012) during the two-choice prediction task.

Five specific hypotheses were tested in this investigation. First, it was hypothesized that if attenuated insular and frontocingulate activations are markers of stimulant addiction, individuals who had recently transitioned to problems with stimulant use (abuse and/or dependence) would exhibit lower activation in these regions during decision making than past occasional stimulant users and stimulant naïve individuals, given research using the two-choice prediction task in amphetamine dependent patients (Paulus et al., 2002b, 2003, 2005). Second, with respect to the role of interoceptive processing alone and its interaction with decision making, it was predicted that current stimulant users will exhibit insular, thalamic, ACC, and PFC attenuation during aversive interoceptive stimuli, consistent with findings in stimulant dependent subjects during the experience of pleasant interoceptive stimuli (May et al., 2013). Third, we forecasted an additive effect of condition and error rate findings, wherein problem stimulant users would show the lowest insula, ACC, and PFC activation compared to former stimulant users and stimulant-naïve subjects during decisions made under uncertainty paired with the aversive interoceptive manipulation. Fourth, given the proposed role of aversive interoceptive stimuli in the maintenance and/or exacerbation of addiction (Paulus et al., 2009; Naqvi and Bechara, 2010; Verdejo-Garcia et al., 2012a), it was hypothesized that problem users would subjectively report higher unpleasantness ratings of the aversive interoceptive stimuli than the other two groups. Fifth, it was predicted that current problem stimulant users would exhibit higher win-stay behavioral responses consistent with previous research in chronic stimulant dependent individuals (Paulus et al., 2002b, 2003). In addition to analyses examining these a-priori hypotheses, given that personality traits linked to addiction such as impulsivity, sensation seeking, and depression are thought to moderate neural mechanisms involved in interoception and decision making (Leland et al., 2006; Paulus et al., 2008, 2009; Verdejo-Garcia et al., 2008; Brewer et al., 2010; Naqvi and Bechara, 2010), exploratory correlations were performed between brain regions of interest (insula, ACC, PFC, thalamus) and personality measures more highly endorsed in current problem stimulant users than past stimulant users and/or stimulant naïve individuals.

## Materials and methods

### Subject recruitment and procedure

The study protocol was approved by the local Human Subjects Review Board (University of California, San Diego) and was carried out in accordance with the Declaration of Helsinki. Individuals were informed that this study was aimed to examine brain functioning of people who use stimulants, and all subjects gave written informed consent. Recreational, non-dependent male and female stimulant users were recruited and defined by methods described in previous experiments involving this sample (Reske et al., 2011; Stewart et al., 2013). Among this original cohort of 184 subjects, these individuals were contacted 3 years after their initial lab visit, with an overall follow-up rate of 93% (171 followed up; 10 unreachable; 3 refused to participate). Each individual underwent a standardized interview during the follow-up assessment to examine the extent of drug use, allowing us to identify subjects in this cohort who developed problems associated with stimulant use and others who had desisted using stimulants. Thus, two stimulant user groups were formed for the present study, termed problem stimulant users (PSU) and desisted stimulant users (DSU).

**Figure 1:**
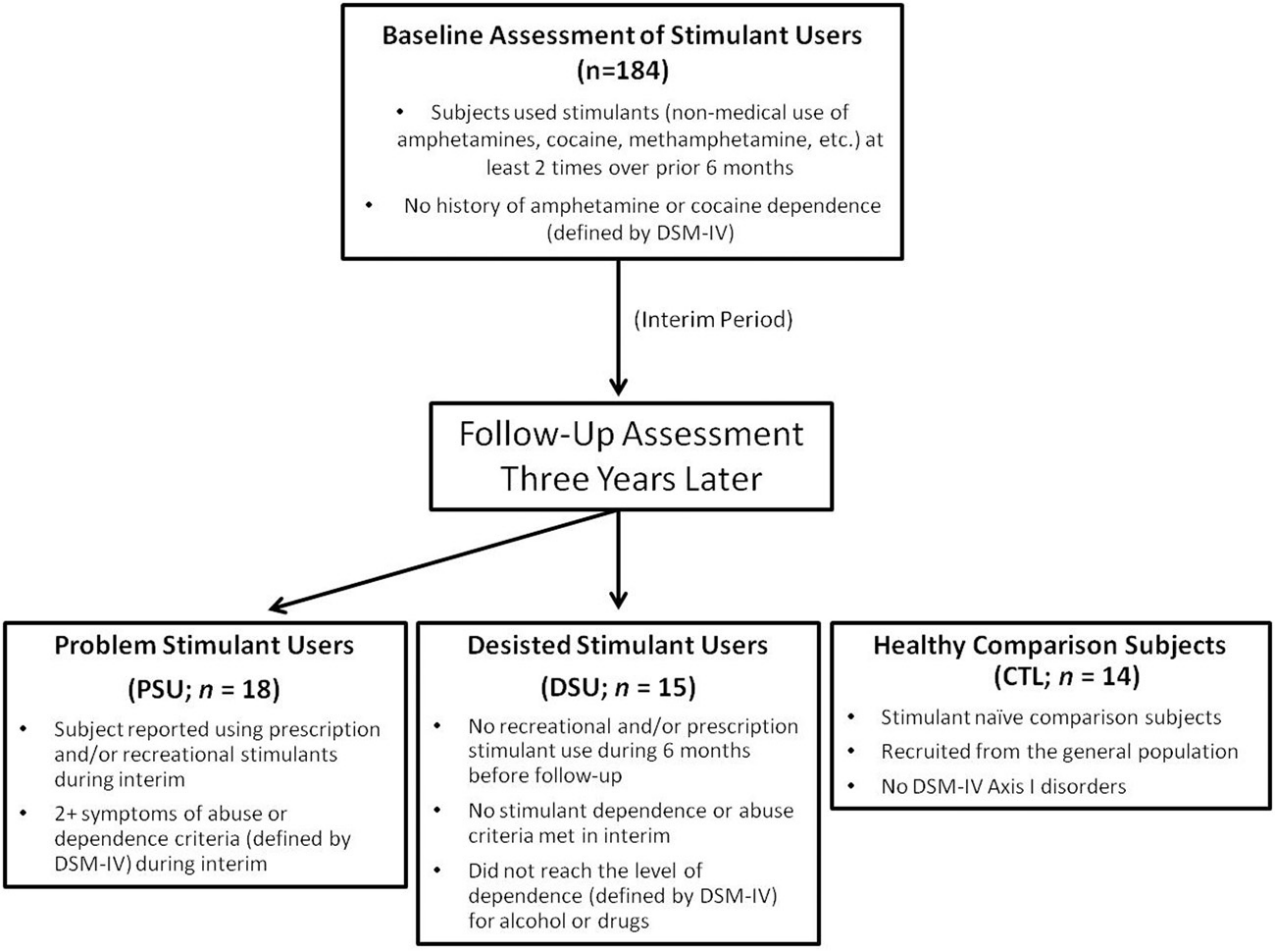
**Timeline of subject recruitment**. Occasional stimulant users were followed up 3 years later to determine which individuals escalated stimulant use (Problem Stimulant Users; PSU) or desisted stimulant use (Desisted Stimulant Users; DSU). Age and education-matched stimulant naIve healthy comparison subjects (CTL) were also recruited.

Specifically, PSU were a priori defined by: (1) continued use of prescription and/or recreational stimulants (e.g., dextroamphetamine, cocaine, methylphenidate) since the initial visit and (2) endorsement of 2+ symptoms of DSM-IV-TR amphetamine and/or cocaine abuse or dependence criteria (American Psychiatric Association, 2000) as defined by the Semi Structured Assessment for the Genetics of Alcoholism II (SSAGA II) (Bucholz et al., 1994) occurring together during at least 6 contiguous months since the initial visit (*M* = 4.78 symptoms; *SD* = 2.24). In comparison, DSU were defined as having: (1) no 6-month periods of time with 3+ uses of reported prescription and/or recreational stimulants, and (2) no endorsement of symptoms of stimulant abuse or dependence (other than nicotine) in the interim as defined by SSAGA II. CTL were recruited from the general population and endorsed no lifetime history of substance or alcohol related problems as determined by SSAGA II (see Figure 1 for schematic overview; see Section Clinical Interview Session for exclusion criteria for each group). Participants from all three groups were selected to be matched on gender, age, and education. No subjects from any group were regular nicotine smokers. The final cohort of the present study (see Table 1) consisted of 18 PSU, 15 DSU, and 14 CTL subjects, all right handed as assessed with the Edinburgh Handedness Inventory (Oldfield, 1971). Subjects then completed two sessions: (1) a clinical interview and questionnaire session; and (2) an fMRI session wherein they completed the two-choice prediction task with breathing load (described below).

**Table 1:** **Group differences in demographics, personality, and drug use**.

	**PSU (9M, 9F)**		**DSU (8M, 7F)**		**CTL (8M, 6F)**		**Group statistics**	
	***M***	***SD***	***M***	***SD***	***M***	***SD***	***F/t/*χ^2^**	***P***
**DEMOGRAPHICS**								
Age (years)	24.39	1.50	24.33	1.54	24.36	2.24	*F*_(2, 44)_ = 0.004	0.99
Education (years)	15.67	1.03	15.53	1.46	16.21	1.37	*F*_(2, 44)_ = 1.15	0.33
WTAR Verbal IQ	110.71	8.30	111.60	8.06	117.82	7.44	*F*_(2, 40)_ = 2.91	0.07
**PERSONALITY MEASURES/PHYSIOLOGY**								
SSS-V Thrill and adventure seeking	6.80	2.18	6.29	2.59	4.82	2.82	*F*_(2, 37)_ = 2.06	0.14
SSS-V Experience seeking	3.07	2.15	2.93	1.49	2.64	1.86	*F*_(2, 37)_ = 0.17	0.84
SSS-V Disinhibition	7.80	2.01	7.36	2.21	8.09	2.63	*F*_(2, 37)_ = 0.34	0.72
SSS-V Boredom susceptibility	7.00	2.04	7.21	1.19	6.00	2.00	*F*_(2, 37)_ = 1.56	0.22
SSS-V Sensation seeking total	24.67	4.89	23.79	6.09	21.55	7.63	*F*_(2, 37)_ = 0.84	0.44
BIS-11 Inattention	11.47	3.09	9.36	2.02	8.64	2.29	*F*_(2, 37)_ = 4.51	0.02
BIS-11 Motor	15.27	2.87	14.14	2.03	17.13	4.16	*F*_(2, 37)_ = 3.24	0.05
BIS-11 Self control	12.73	2.43	12.07	2.90	12.09	3.81	*F*_(2, 37)_ = 0.22	0.80
BIS-11 Cognitive complexity	11.73	3.31	10.29	2.05	11.45	1.75	*F*_(2, 37)_ = 1.29	0.29
BIS-11 Perseverence	7.33	1.80	7.93	2.34	8.09	2.26	*F*_(2, 37)_ = 0.48	0.62
BIS-11 Cognitive instability	6.27	1.83	6.00	1.36	5.45	2.02	*F*_(2, 37)_ = 0.70	0.50
BIS-11 Impulsivity total	66.67	12.66	59.79	7.32	61.00	9.57	*F*_(2, 37)_ = 1.86	0.17
BDI-II Depression total	5.50	6.62	0.87	1.30	2.57	2.87	*F*_(2, 44)_ = 4.58^a^	0.02
STAI Trait anxiety	35.22	8.25	29.87	4.96	35.64	7.57	*F*_(2, 44)_ = 3.08^a^	0.06
STAI State anxiety	30.94	7.71	26.13	5.66	29.56	5.08	*F*_(2, 44)_ = 2.40	0.10
BPQ Body perception awareness	2.36	1.18	2.67	0.93	2.40	1.13	*F*_(2, 37)_ = 0.40	0.72
BPQ Stress response	2.43	1.08	2.66	0.99	2.58	1.01	*F*_(2, 37)_ = 0.19	0.83
BPQ Body perception ANS reactivity	1.31	0.29	1.54	0.45	1.20	0.22	*F*_(2, 36)_ = 3.20	0.05
BPQ Stress style 1	2.63	0.68	2.81	0.38	2.54	0.68	*F*_(2, 37)_ = 0.73	0.49
BPQ Stress style 2	1.45	0.55	1.61	0.48	1.18	0.32	*F*_(2, 37)_ = 2.52	0.09
Average CO_2_	1.37	0.30	1.28	0.19	1.37	0.32	*F*_(2, 29)_ = 0.44	0.65^†^
**DRUG USE AND CRAVING**								
Stimulant craving desire to use	15.23	8.72	12.36	3.75	–	–	*t*_(25)_ = 1.13	0.27
Stimulant craving plan to use	21.23	7.93	18.82	4.88	–	–	*t*_(25)_ = 0.96	0.35
Stimulant craving anticipate positive outcome	27.92	7.18	24.89	10.48	–	–	*t*_(25)_ = 0.87	0.39
Stimulant craving anticipate relief from withdrawal	27.88	6.56	25.07	8.65	–	–	*t*_(25)_ = 0.95	0.35
Stimulant craving lack of control over use	16.38	9.15	14.29	7.50	–	–	*t*_(25)_ = 0.65	0.52
Amphetamine and cocaine uses as of initial visit (# sessions)	86.11	100.50	32.80	49.67	–	–	*t*_(31)_ = 1.98^a^	0.06
Interim amphetamine and cocaine uses (# sessions)	752.06	1223.33	7.80	16.92	–	–	*t*_(31)_ = 2.58^a^	0.02
Lifetime marijuana uses	2168.06	3945.17	1550.93	2154.19	16.07	28.50	*F*_(2, 44)_ = 2.51^a^	0.09
(# sessions)							*t*_(31)_ = 0.54	0.59
**DSM-IV Abuse/dependence diagnoses**	**Percentage**		**Percentage**		**Percentage**			
Current alcohol abuse	61		47		29		χ^2^_(1)_ = 3.34^b^	0.18
Current alcohol dependence	17		7		0		χ^2^_(1)_ = 0.77^c^	0.38
Current marijuana abuse	50		67		7		χ^2^_(1)_ = 0.93^c^	0.34
Current marijuana dependence	17		0		0		–	–
Current amphetamine abuse	56		0		0		–	–
Current cocaine abuse	56		0		0		–	–
Current amphetamine dependence	28		0		0		–	–
Current cocaine dependence	28		0		0		–	–

a Group variances are unequal.

b Test compared all three groups.

c Test compared PSU and DSU only.

† Group main effect from CO_2_ repeated measures analysis of variance. PSU, Problem Stimulant Users. DSU, Desisted Stimulant Users. CTL, Healthy Comparison Subjects. WTAR, Wechsler Test of Adult Reading. SSS-V, Sensation Seeking Scale. BIS-11, Barratt Impulsivity Scale. BDI-II, Beck Depression Inventory II. STAI, State-Trait Anxiety Inventory. BPQ, Body Perception Questionnaire. ANS, Autonomic Nervous System. Stimulant Craving Questionnaire, average of subjects' responses to the Cocaine Craving Questionnaire (CCQ) filled out twice, once with respect to amphetamine use and once with respect to cocaine use. CO_2_, carbon dioxide. DSM-IV, Diagnostic and Statistical Manual of Mental Disorders IV. The following number of subjects did not complete specific measures: WTAR: n = 1 PSU and n = 3 CTL; SSS-V, BPQ, and BIS-11: n = 3 PSU, n = 1 DSU, n = 3 CTL; Stimulant Craving (CCQ): n = 6 PSU, n = 1 DSU. An additional CTL was an outlier (>3SD from mean) on BPQ ANS Reactivity and therefore was not included in analysis of that particular subscale.

### Clinical interview session

Subjects were assessed by experienced interviewers using the SSAGA II and diagnoses were based on consensus meetings (accredited clinician Martin P. Paulus and trained study personnel). The following were exclusion criteria for all groups: (1) incorporated metal or any other factor that precludes use of fMRI; (2) head injuries or loss of consciousness for longer than 5 min; (3) prescription medication for attention deficit hyperactivity disorder (ADHD), depression, bipolar disorder, anxiety and other psychiatric disorders taken currently and/or within the past 3 years; (4) any diagnosed neurological disorder (including ADHD); (5) evidence for lifetime psychosis (e.g., schizophrenia, bipolar disorder) or antisocial personality disorder; (6) current and/or past 6 month episodes of DSM-IV anxiety disorders or unipolar depression; and (7) a positive urine toxicology screen for any substance other than marijuana at the time of the fMRI session (given that marijuana can be present in urine as long as 6 weeks after use).

At the time of the clinical interview, several personality and symptom assessment questionnaires known to correlate with substance use disorders were administered, including the Sensation Seeking Scale (SSS) (Zuckerman, 2007), the Barratt Impulsiveness Scale (BIS-11) (Patton et al., 1995), the State-Trait Anxiety Inventory (STAI) (Spielberger et al., 1983), and the Beck Depression Inventory II (BDI-II) (Beck et al., 1996). To assess trait interoceptive responses to stress, subjects completed the Body Perception Questionnaire (BPQ) (Porges, 1993). In addition, PSU and DSU completed the Cocaine Craving Questionnaire (CCQ) (Tiffany et al., 1993) twice, once with respect to craving linked to amphetamine use and once with respect to craving linked to cocaine use.

### fMRI session

All subjects were required to abstain from drugs for 72 h prior to the fMRI session. Two subjects tested positive for marijuana on the pre-fMRI urine toxicology screen (*n* = 1 PSU; *n* = 1 DSU) but no subjects tested positive for any other substances.

#### Breathing load apparatus

During the entire fMRI session, subjects wore a nose clip and respired through a mouthpiece and non-rebreathing valve (2600 series, Hans Rudolph). The apparatus was attached to the fMRI scanner head coil to eliminate the need for the subject to contract mouth muscles while maintaining an airtight seal. The resistance load was a stainless steel screen mesh disk placed in a Plexiglas tube (loading manifold). Subjects were given a 40 cmH_2_O/L/s inspiratory load applied to only the inspiratory port of the non-rebreathing valve for 40 s at a time. Prior to scanning, subjects were given instructions about the task and experienced three 1-min segments of the breathing load. After the fMRI session, subjects completed Visual Analog Scale (VAS) questionnaires, on which they were asked to rate the breathing load experience on a 10 cm scale anchored from “not at all” (0) to “extremely” (10) on the following 16 dimensions: pleasant, unpleasant, intense, tingling, fear of losing control, faintness, fear of dying, unreality, hot/cold flushes, trembling, choking, abdominal distress, chest pain, palpitations, sweating, and dizziness, corresponding to items used in prior studies (Chan and Davenport, 2008; Davenport and Vovk, 2008).

#### Two-choice prediction task with breathing load manipulation

**Figure 2:**
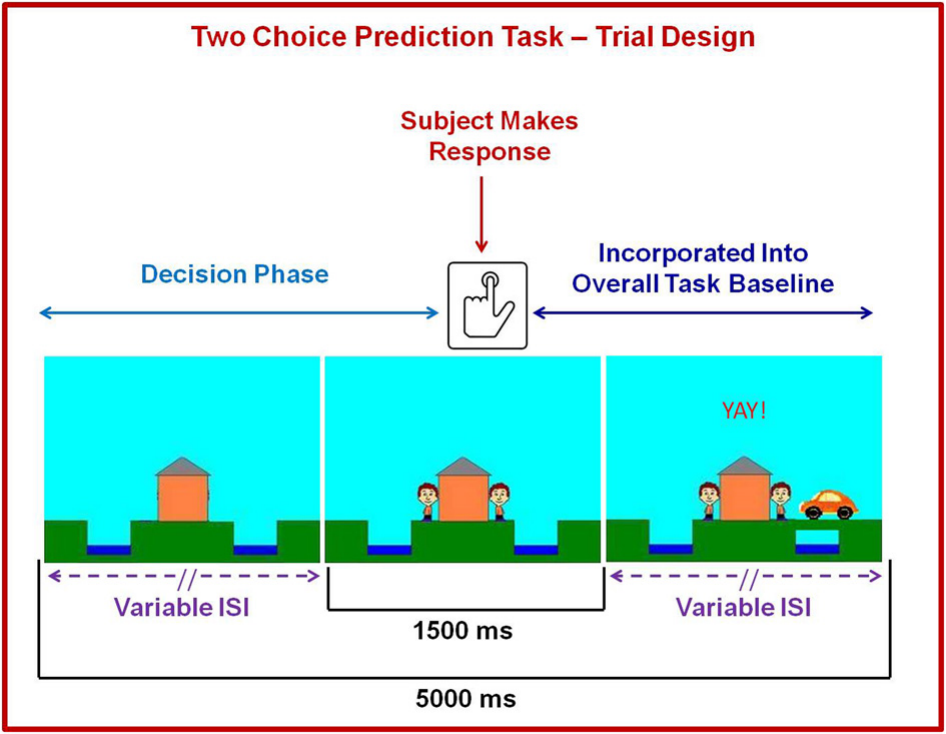
**Illustration of two choice prediction task**. For each trial, a house was shown in the center with two people: one on the left and one on the right of the house. Subjects pressed a button to predict whether a car would come by on the left or right side to pick up the person. After the subject made a decision, the car was presented on the left or right side of the screen. If the selected response matched the side where the car was presented, the person on the selected side met up with the car. Although each trial lasted 5000 ms and subjects were allowed to respond with a button press during a fixed 1500 ms period at the point when they saw the two people on the screen, the length of the beginning and ends of each trial were designed to have variable interstimulus intervals (lSI). Brain activation consisting of time from trial onset to the button press was included in fMRI analysis as a decision regressor of interest, wherein brain activation during the remainder of the trial was incorporated into the overall baseline regressor.

The two-choice prediction task has been utilized to determine the response characteristics in decision making situations with uncertain outcomes (Paulus et al., 2002b, 2003, 2005). The version of the two-choice prediction task employed in the present study also included an aversive interoceptive breathing load manipulation. Figure 2 shows that for each trial (lasting for a fixed duration of 5000 ms), a house was shown in the center of the computer screen (variable duration: 416–797 ms), followed by an updated image of the house with two people: one to the left and one to the right (fixed duration of 1500 ms). Subjects were told that, as soon as they saw two people appear next to the house, their task was to predict whether a car would come by to pick up the person on the left or right side of the computer screen by pressing a left or right button, respectively. Subjects had 1500 ms to register a response. If subjects did not respond during a particular trial, they automatically received negative feedback following response timeout. Participants were given no predictive information and had to make a choice based on the history of preceding responses and outcomes. After the 1500 ms reaction time window ended, the car was presented on the far left or right side of the screen for the remainder of the trial (variable duration: 2703–3084 ms). If the selected response (left or right) matched the side where the car was presented, the person on the selected side met up with the car. Unbeknownst to the subject, the car was presented according to a predetermined schedule. Specifically, a computer algorithm, which took each subject's response into account, determined whether a response would be “correct” or “incorrect.” Correct responses consisted of the word “YAY!” presented in the top center of the computer screen for the remainder of the trial duration, while incorrect responses consisted of the word “BOO!” presented in the same location. The time of trial onset to the subject's button press on each trial was considered the decision phase of interest during the task, whereas the remaining portion of the trial was incorporated into the overall baseline with which the decision phase was later compared.

**Figure 3:**
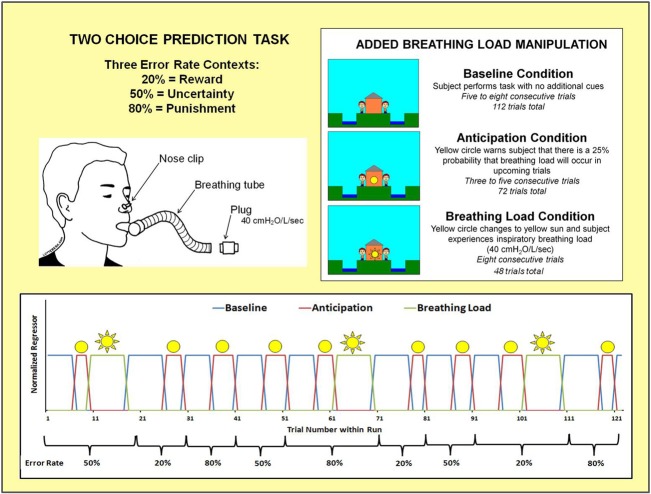
**Illustration of two choice prediction task with added breathing load manipulation**. Unbeknownst to subjects, the task was divided into three blocks of trials with differing reinforcement schedules: 20, 50, and 80% error rates. Within the context of each error rate, subjects also experienced three interoception conditions: baseline, anticipation of breathing load, and experience of breathing load.

Figure 3 shows that the two-choice prediction task was divided into three types of trials with differing reinforcement, or error, rates: (1) 20% *error rate*, indexing response to reward, wherein “YAY” feedback is presented on the computer screen after 80% of each subject's responses and “BOO” feedback is presented on the computer screen for the remaining 20% of trials; (2) 50% *error rate*, indexing response to uncertainty, wherein “YAY” feedback is presented after 50% of each subject's responses and “BOO” feedback is presented for the remaining 50% of trials; and (3) 80% *error rate*, indexing response to punishment, wherein “YAY” feedback is presented after 20% of each subject's responses and “BOO” feedback is presented for the remaining 80% of trials. Two runs of 122 trials each were presented to subjects (total number of trials presented for each error rate: 20% = 80, 50% = 84, 80% = 80). Each error trial type was presented consecutively for 9–20 trials.

Within the context of each error rate, subjects also experienced an aversive interoceptive manipulation, involving three conditions: (1) *baseline* (ranging from 6 to 8 consecutive trials): no additional cues are presented on the screen; (2) *anticipation* (ranging from 3 to 5 consecutive trials): a yellow circle shown in the center of the house, warning the subject that there was a 25% chance that their breathing would be loaded in upcoming trials; and (3) *breathing load* (8 consecutive trials): a yellow sun shown in the center of the house, wherein subject experienced an inspiratory 40 cmH_2_O/L/s breathing load for 40 s duration. This paradigm was implemented using an event-related fMRI design, consisting of 2 runs with 306 repetition times (TR) each (*TR* = 2000 ms; 2.5 TR per trial). The total number of trials presented for baseline, anticipation, and breathing load interoception conditions were 112, 72, and 48, respectively, with a total of 24 anticipation and 16 breathing load trials presented for each of the three error rates. The remaining 12 trials were null trials distributed across the three error types. At least 3 consecutive trials were presented for baseline and anticipation conditions. The breathing load condition always consisted of 8 trials. Response latency and button choice (left, right) were recorded for each trial. The order of conditions and error rates (see Figure 3) were kept fixed across subjects, although the specific feedback given to each subject within each error rate context was contingent upon frequency of their responses in order to match up with the reinforcement determined by each error rate.

### Neuroimaging acquisition and analysis

Images were acquired using a 3T GE CXK4 Magnet at the UCSD Center for Functional MRI, which is equipped with 8 high-bandwidth receivers that allow for shorter readout times and reduced signal distortions and ventromedial signal dropout. Each 1-h session included: (1) a standard anatomical protocol consisting of a spoiled gradient recalled (SPGR) sequence (FOV 25.6 cm; 192 × 256 matrix; 172 sagittally acquired slices of 1 mm thickness; *TR*: 8 ms; *TE*: 3 ms; flip angle = 12) and (2) two runs of the two-choice prediction task (for each run: axial T2^*^-weighted echo-planar images (EPI); FOV 24 cm; 64 × 64 matrix; 40 slices of 3 mm thickness; 1.4 mm gap; *TR* = 2000 ms; *TE* = 30 ms; flip angle = 90°; first two TRs were discarded to allow for BOLD signal stabilization). During the two-choice prediction task, carbon dioxide (CO_2_) levels were also collected at a rate of 40 Hz for each subject via nasal cannula.

#### First level analysis

All subject-level structural and functional image processing was computed with the Analysis of Functional Neuroimages (AFNI) software package (Cox, 1996). The multivariate regressor approach detailed below was used to relate changes in EPI intensity to differences in task characteristics (Haxby et al., 2000). EPI images were co-registered using a 3D-coregistration algorithm (Eddy et al., 1996) that was developed to minimize the amount of image translation and rotation relative to all other images. Six motion parameters (dx, dy, dz, and roll, pitch, and yaw) were obtained across the time series for each subject and the latter three were used as regressors to adjust EPI intensity changes due to motion artifacts. This has been shown to increase power in detecting task-related activation (Skudlarski et al., 1999). Slice timing correction was then performed, followed by automatic coregistration of the EPI to the high-resolution anatomical image. Each dataset was manually inspected to confirm successful alignment. New outliers were generated for the volume-registered dataset using AFNI's 3dToutcount. If > 10% voxels were marked as outliers within a particular TR that time point was then excluded from further analysis. Approximately 1% of TRs were censored (across subjects for entire task: *M* = 7.15, *SD* = 4.89, range = 0–21).

Nine decision regressors of interest were generated to delineate trials with differing error rates (20%, 50%, 80%) and conditions (baseline, anticipation, breathing load), with timing of the decision phase for each regressor based on individual subjects' reaction times during each trial (see Figure 2). These regressors were convolved with a gamma variate function for each subject using the AFNI waver program (Boynton et al., 1996) to model a prototypical hemodynamic response consisting of a 6–8 s delay (Friston et al., 1995) and account for temporal dynamics of the hemodynamic response (typically 12–16 s). All nine convolved time series were normalized. Three movement regressors (roll, pitch, yaw), an overall non-decision related task baseline regressor (see Figure 3), a linear drift regressor computed by AFNI, and the nine decision making regressors (20% error: baseline [*n* = 36 trials], anticipation [*n* = 24 trials], and breathing load [*n* = 16 trials]; 50% error: baseline [*n* = 40 trials], anticipation [*n* = 24 trials], breathing load [*n* = 16 trials], and 80% error: baseline [*n* = 36 trials], anticipation [*n* = 24 trials], and breathing load [*n* = 16 trials]) were included in a linear regression model in AFNI's 3dDeconvolve program to estimate the goodness of fit between model estimates and BOLD responses for each subject. Following the deconvolution, voxels were resampled into 4 × 4 × 4 mm^3^ space and whole-brain voxel-wise normalized percent signal change, the main dependent measure, was determined by dividing the beta coefficient for each of the nine decision predictors by the beta coefficient for the non-decision related overall baseline regressor and multiplying by 100. Next, a Gaussian spatial filter (4 mm full width half maximum) was used to spatially blur percent signal change values to account for anatomical differences and this output was then normalized to Talairach coordinates (40 × 48 × 38 voxel coverage) as defined by AFNI's built-in atlases. Finally, individual subject percent signal change scaled beta weight values for error rates and interoception conditions (baseline 20%, baseline 50%, baseline 80%, anticipation 20%, anticipation 50%, anticipation 80%, breathing load 20%, breathing load 50%, and breathing load 80%) were extracted for their use as dependent measures in group analyses.

#### Second level analysis

A linear mixed effects (LME) model (Pinheiro et al., 2013) was computed in R (R-Development-Core-Team, 2008) for each voxel, wherein group (PSU, DSU, CTL), error rate (20%, 50%, 80%), and interoception condition (baseline, anticipation, breathing load) were modeled as fixed factors, whereas subject was modeled as a random factor. Percent signal change scaled beta weight value was the dependent variable. For each voxel, degrees of freedom, *F*, and *p*-values were obtained for each main effect and interaction. Next, significant clusters of voxels were extracted using a threshold adjustment method based on 1000 Monte-Carlo simulations (AFNI's program Alpha Sim), which guarded against identifying false positive areas of activation (considering whole brain voxel size, 4 mm smoothness). For main effects and interactions involving group, AlphaSim identified a minimum cluster volume of 512 μL (8 contiguous voxels) to result in a voxel-wise probability of *p* < 0.02 significance (*p* < 0.01 two tailed), corrected for multiple comparisons. The voxelwise threshold for effects of interest were based on the following LME degrees of freedom and *F* values: (1) Group main effect: *F*_(2, 44)_ = 4.28; (2) Error rate and interoception condition main effects: *F*_(2, 352)_ = 3.95; (3) Group by error rate, group by condition, and error rate by interoception condition interactions: *F*_(4, 352)_ = 2.96; and (4) Group by error rate by interoception condition interaction: *F*_(8, 352)_ = 2.31^1^.

### Questionnaire/interview analysis

Group differences in demographics, personality measures, and state emotion were evaluated using Predictive Analytics Software (PASW) (SPSS, 2009) univariate analysis of variance (ANOVA) and Bonferroni *post-hoc* tests for significant results. In addition, *t*-tests were used to examine differences between PSU and DSU in stimulant craving and interim/lifetime drug use (number of distinct sessions used) and chi-square tests compared frequency of substance abuse/dependence diagnoses between groups.

### Physiological analysis

CO_2_ data were visually inspected for artifacts and down sampled by 80 (40 Hz * 2 s per TR) to obtain one value per TR per fMRI run. A total of 32/47 (68%) of subjects (*n* = 11 DSU and CTL; *n* = 10 PSU) had usable CO_2_ data for both runs. For these subjects, CO_2_ values during each error rate and interoception condition were extracted, averaged, and input into a repeated measures ANOVA with condition and error rate as within-subjects factors, and group as the between-subjects factor.

### Behavioral analysis

A LME was computed in R for percentage of win-stay responses, wherein group (PSU, DSU, CTL) and error rate (20%, 50%, 80%) were fixed factors and subject was modeled as a random factor. Condition was not included as a factor because the anticipation condition consisted of too few trials within each error rate to extract reliable probability estimates.

### Exploratory analysis

Given that PSU endorsed higher BIS inattention-related impulsivity, BDI-II depression, and higher number of interim stimulant uses (the latter two natural log transformed due to non-normality) than DSU and/or CTL (please see results below), within the PSU group correlations were computed between each of these three measures and variables of interest in order to assist in the explanation of results: (1) percentage of win-stay responses averaged across error rates; (2) VAS unpleasantness ratings; and (3) thalamus, PFC, ACC, and insula activation emerging as significant from LME results. Correlations were then corrected for multiple comparisons (*p* = 0.05/18 = 0.003).

## Results

### Questionnaire/interview analysis

Demographic, personality, state emotion, and drug use information is presented in Table 1. Groups were comparable in age and education and endorsed similar levels of sensation seeking, anxiety, and levels of alcohol abuse. PSU endorsed higher levels of BDI-II depression than DSU (*post-hoc p* = 0.01) but neither group differed from CTL. PSU also reported higher BIS Inattention scores than CTL (*post-hoc p* = 0.02) but both groups did not differ from DSU. With respect to drug use, PSU and DSU reported similar levels of current stimulant craving, current marijuana abuse, current alcohol abuse and dependence, and lifetime marijuana use. However, PSU endorsed greater stimulant use in the 3-year interim period prior to the interview/fMRI sessions than DSU as well as higher levels of current stimulant abuse and stimulant and marijuana dependence.

### Physiological analysis

A main effect of interoception condition emerged [*F*_(2, 58)_ = 26.82, *p* < 0.001], wherein breathing load was associated with lower CO_2_ (*M* = 1.22, *SE* = 0.05) than baseline (*M* = 1.40, *SE* = 0.05) and anticipation (*M* = 1.41, *SE* = 0.05) across subjects. No other main effects or interactions, including those with group, approached significance (all *p* > 0.29).

### VAS analysis

**Figure 4:**
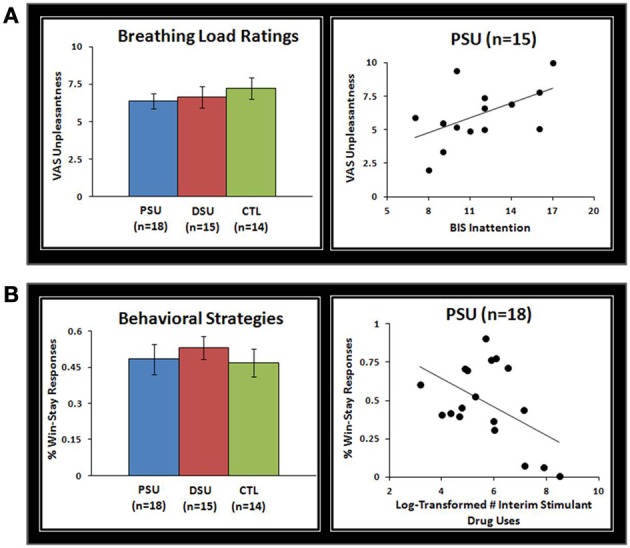
**(A)** Although problem stimulant users (PSU), desisted stimulant users (DSU), and healthy comparison subjects (CTL) did not differ on visual analogue scale (VAS) unpleasantness ratings of the breathing load stimuli, within PSU higher Barratt Impulsivity Scale (BlS) Inattention scores were associated with higher VAS unpleasantness ratings. Three PSU subjects did not have BIS scores and therefore were not included in scatterplots. **(B)** Although groups did not differ in percentage of win-stay responses across error-rates, within PSU a greater frequency of stimulant drug uses in the past 3 year interim period was associated with a lower percentage of win-stay responses. Error bars indicate ± 1 standard error.

Subjective ratings of the aversive interoceptive manipulation are presented in Table 2. Overall, across subjects the breathing load stimulus was rated low in pleasantness, moderately high in unpleasantness, and moderate in intensity. Although groups did not differ on any of the sixteen dimensions rated, exploratory analyses (Figure 4A) showed that within PSU, higher BIS Inattention scores were associated with greater unpleasantness ratings of the breathing load stimuli (*r* = 0.54, *p* = 0.04), although this correlation did not survive correction for multiple comparisons.

**Table 2:** **Post-fMRI Visual Analog Scale (VAS) ratings of aversive interoceptive stimulus (breathing load)**.

	**PSU (*n* = 18)**		**DSU (*n* = 15)**		**CTL (*n* = 14)**		**Group statistics**	
	***M***	***SD***	***M***	***SD***	***M***	***SD***	***F*_(2, 44)_**	***p***
Pleasantness	1.54	1.90	1.24	1.45	0.91	0.90	0.68	0.51
Unpleasantness	6.37	2.14	6.65	2.65	7.21	2.62	0.46	0.63
Intensity	3.99	2.51	3.65	2.45	4.33	3.56	0.21	0.82
Tingling sensations	1.18	1.80	1.37	2.23	0.55	1.16	0.82	0.45
Fear of losing control	0.98	1.67	0.93	1.52	1.55	2.57	0.46	0.63
Faintness	1.91	2.39	0.91	0.92	1.19	2.12	1.16	0.32
Fear of dying	0.55	1.01	0.27	0.79	0.21	0.42	0.85	0.44
Unreality	0.88	1.84	0.63	1.46	0.23	0.39	0.82	0.45
Hot/cold flashes	0.67	1.45	0.41	0.81	0.59	1.97	0.13	0.88
Trembling	0.96	1.73	0.37	1.04	0.17	0.39	1.78	0.18
Choking	0.83	1.72	0.57	1.13	1.04	2.48	0.23	0.79
Fear of going crazy	1.08	2.04	0.21	0.52	0.16	0.29	2.58	0.09
Abdominal distress	0.38	0.75	0.62	1.20	0.24	0.45	0.73	0.49
Chest pain	0.71	1.47	0.50	0.96	0.26	0.54	0.63	0.54
Palpitations	0.77	1.77	0.27	0.57	0.93	1.99	0.69	0.51
Sweating	0.67	1.34	0.31	0.58	0.24	0.39	1.07	0.35
Dizziness	1.94	2.61	1.11	1.14	1.02	2.37	0.90	0.41

PSU, Problem Stimulant Users; DSU, Desisted Stimulant Users; CTL, Healthy Comparison Subjects; VAS scales range from 0–10.

### Behavioral analysis

Results indicated that group main effects or interactions with error rate did not emerge for win-stay responses (*p* = 0.43 and *p* = 0.45). However, an error rate main effect was evident for win-stay responses, which were more frequent across subjects for 20% error rate (54%) than 80% error rate (46%) [*F*_(2, 86)_ = 7.8, *p* = 0.001]. Despite no group differences in win-stay behavior, Figure 4B illustrates that within PSU higher interim stimulant use was associated with lower rates of win-stay behavior during the task (*r* = −0.49, *p* = 0.04), although this finding did not survive correction for multiple comparisons.

### fMRI analysis

No significant results emerged for the group main effect, the error rate by interoception condition interaction, or the group by error rate by interoception condition interaction using the voxelwise corrected *p* = 0.02 threshold^2^.

#### Error rate main effect

**Figure 5:**
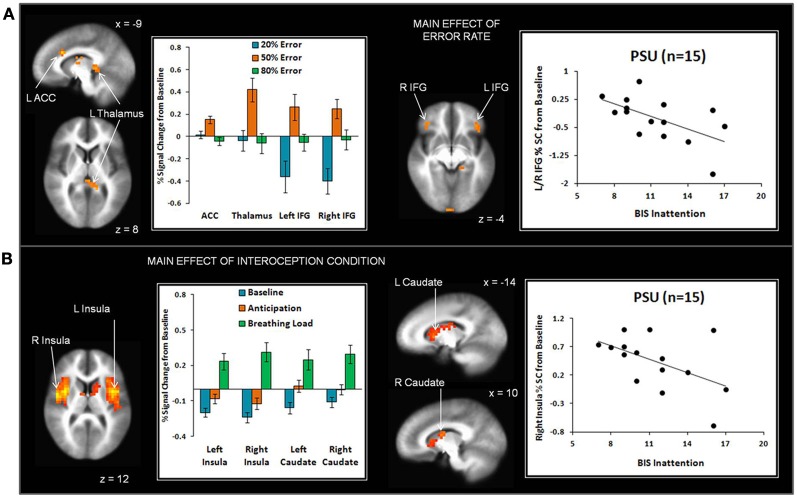
**(A)** The main effect of error rate indicates that uncertainty (50% error rate) elicited greater anterior cingulate cortex (ACC), thalamus, and inferior frontal gyrus (IFG) activation than reward (20% error rate) and punishment (80% error rate). Within the problem stimulant user (PSU) group, higher Barratt Impulsivity (BIS) Inattention scores were associated with lower thalamus and IFG activation in response to 20% error rate. **(B)** The main effect of interoception condition shows that breathing load elicited greater anterior and posterior insula and dorsal striatum (caudate) activation than baseline and anticipation conditions. Within the PSU group, higher BIS Attention scores were linked to lower right insula activation during breathing load. Three PSU subjects did not have BIS scores and therefore were not included in scatterplots. Error bars indicate ± 1 standard error.

Across subjects, the 50% error rate was associated with greater left dorsal ACC, left thalamus, bilateral inferior frontal gyrus (IFG), and right inferior temporal gyrus activation than the 20% and 80% error rates (see Figure 5A and Table 3). In addition, the 80% error rate resulted in greater bilateral IFG activation than the 20% error rate. Exploratory analyses revealed that within PSU, higher BIS Inattention scores were associated with lower left thalamus and bilateral IFG activation in response to the 20% error rate (thalamus *r* = −0.53, *p* = 0.04; left IFG *r* = −0.55, *p* = 0.03; right IFG *r* = −0.57, *p* = 0.03), although both findings did not survive correction for multiple comparisons.

**Table 3:** **fMRI Results for the main effect of error rate**.

**Volume (μL)**	**No. of voxels in cluster**	***x***	***y***	***z***	**L/R**	**BA**	**Region**
**50% ERROR RATE > 20% ERROR RATE AND 80% ERROR RATE**							
5184	81	−19	3	29	L	24	Cingulate gyrus (including dorsal anterior cingulate)^†^
3200	50	−6	−24	11	L		Thalamus^†^
704	11	7	−96	−8	R	18	Lingual gyrus^†^
640	10	34	−61	−23	R		Culmen
640	10	−49	−22	19	L	13	Postcentral gyrus
512	8	27	−31	−22	R	35	Culmen
**50% ERROR RATE > 80% ERROR RATE > 20% ERROR RATE**							
896	14	−29	15	−14	L	47	Inferior frontal gyrus
832	13	37	29	−8	R	47	Inferior frontal gyrus
768	12	−40	29	−7	L	47	Inferior frontal gyrus
640	10	57	−10	−19	R	20	Inferior temporal gyrus

L, left hemisphere; R, right hemisphere; BA, Brodmann Area; Coordinates reflect center of mass for each cluster. All clusters emerged as greater than F_(2, 352)_ = 3.95, p = 0.02 corrected voxelwise for multiple comparisons (minimum significant cluster = 8 voxels).

† Regions that also remained significant at F_(2, 352)_ = 4.66, p = 0.01 corrected.

#### Interoception condition main effect

Given that conditions differed across a large portion of the cortex, (e.g., 3863 contiguous voxels emerged as significant for the whole-brain analysis), a restricted mask (threshold *p* = 0.02 pcorrected for multiple comparisons) was used to examine differences as a function of breathing load for insula, ACC, striatum, and thalamus, brain regions implicated in the processing of interoception in response to pleasant stimuli (May et al., 2013). Across subjects, the breathing load condition was associated with greater activation in bilateral anterior/posterior insula and bilateral dorsal striatum (caudate) than the anticipation condition, which in turn elicited greater activation than the baseline condition (see Figure 5B and Table 4). Although groups did not differ in insula activation during breathing load, exploratory analyses showed that within PSU, higher BIS Inattention scores were associated with lower right insula activation during breathing load (*r* = −0.52, *p* = 0.049) but this finding did not survive correction for multiple comparisons.

**Table 4:** **fMRI results for the main effect of interoception condition**.

**Volume (μL)**	**No. of voxels in cluster**	***x***	***y***	***z***	**L/R**	**BA**	**Region**
**BREATHING LOAD > ANTICIPATION > BASELINE**							
14912	233	−39	1	9	L	13	Anterior/posterior insula
14528	227	40	−1	11	R	13	Anterior/posterior insula
3840	60	−13	2	13	L	–	Caudate
2112	33	14	−8	19	R	–	Caudate

L, left hemisphere; R, right hemisphere; BA, Brodmann Area. Coordinates reflect center of mass for each cluster. All clusters emerged as greater than F_(2, 352)_ = 3.95, p = 0.02 corrected voxelwise for multiple comparisons (minimum significant cluster = 8 voxels). All regions remained significant at F_(2, 352)_ = 4.66, p = 0.01 corrected.

#### Group by error rate interaction

**Figure 6:**
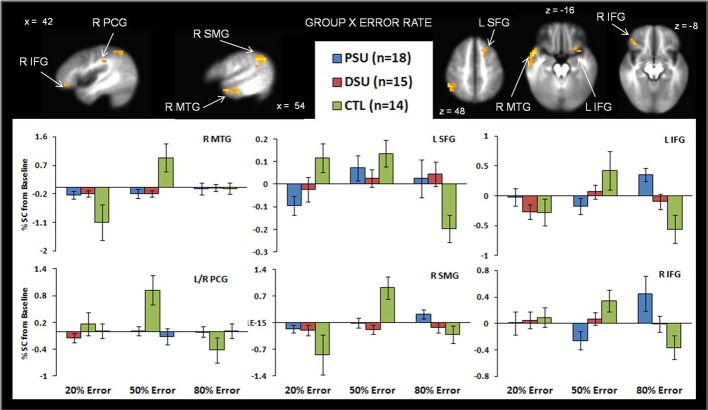
**The group by error rate interaction demonstrates that, compared to healthy comparison subjects (CTL), problem stimulant users (PSU) exhibited (1) lower left middle temporal gyrus (MTG) bilateral postcentral gyrus (PCG), and right supramarginal gyrus (SMG) activation in response to uncertainty (50% error rate); (2) lower left superior frontal gyrus activation in response to reward (20% error rate); and (3) higher bilateral inferior frontal gyrus (IFG) to punishment**. Activation is reflected as percent signal change (%SC) from baseline. Error bars indicate ± 1 standard error.

Table 5 demonstrates that for the 20% error rate, PSU exhibited lower activation than CTL in left superior frontal gyrus (SFG). For the 50% error rate, PSU showed lower activation than CTL in bilateral temporal gyri, bilateral postcentral gyri, right supramarginal gyrus, and right IFG (see Figure 6). In contrast, for the 80% error rate, PSU exhibited greater bilateral IFG activation than DSU and CTL.

**Table 5:** **fMRI results for the group by error rate interaction**.

**Volume (μL)**	**No. of voxels in cluster**	***x***	***y***	***z***	**L/R**	**BA**	**Region**	**20% Error**	**50% Error**	**80% Error**
1984	31	53	−2	−17	R	21	Middle/superior/inferior temporal gyrus^†^	ns	CTL > Other 2	ns
576	9	−6	−43	72	L	5	Postcentral gyrus	ns	CTL > Other 2	ns
512	8	33	−61	−25	R	–	Culmen	ns	CTL > Other 2	ns
512	8	27	−29	−20	R	35	Parahippocampal gyrus	ns	CTL > Other 2	ns
512	8	38	33	−10	R	11/47	Inferior frontal gyrus	ns	PSU < Other 2	PSU > Other 2
512	8	−28	14	−18	L	47	Inferior frontal gyrus	ns	ns	PSU > Other 2
1856	29	55	−47	33	R	40	Supramarginal gyrus^†^	ns	CTL > PSU	PSU > Other 2
640	10	−59	−36	4	L	22	Middle temporal gyrus^†^	ns	CTL > PSU	PSU > Other 2
960	15	45	−51	48	R	40	Inferior parietal lobule^†^	ns	ns	CTL < Other 2
768	12	−20	17	46	L	8	Superior frontal gyrus	CTL > PSU	ns	CTL < Other 2
768	12	23	−81	−28	R	–	Tuber, uvula, pyramis^†^	DSU > Other 2	ns	DSU > Other 2
576	9	43	−27	35	R	2	Postcentral gyrus	ns	PSU < Other 2	ns

L, left hemisphere; R, right hemisphere; BA, Brodmann Area; PSU, Problem Stimulant Users; DSU, Desisted Stimulant Users; CTL, Healthy Comparison Subjects. Other 2, remaining two groups. Coordinates reflect center of mass for each cluster. All clusters emerged as greater than F_(4, 352)_ = 2.96, p = 0.02 corrected voxelwise for multiple comparisons (minimum significant cluster = 8 voxels).

† *Regions that remained significant at F_(4, 352)_ = 3.37, p = 0.01 corrected*.

#### Group by interoception condition interaction

**Figure 7:**
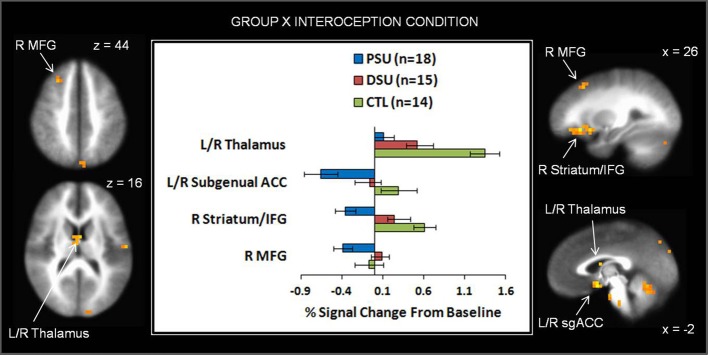
**The group by interoception condition interaction indicates that during the breathing load condition, problem stimulant users (PSU) exhibited lower activation than desisted stimulant users (DSU) and healthy comparison subjects (CTL) in right middle frontal gyrus (MFG), bilateral thalamus, bilateral subgenual anterior cingulate (ACC), right striatum, and right inferior frontal gyrus (IFG)**. Error bars indicate ± 1 standard error.

On the whole, similar patterns of activation emerged for baseline and anticipation conditions as a function of group. However, during the breathing load condition, PSU exhibited lower activations in bilateral subgenual ACC, right striatum, right middle frontal gyrus (MFG), right IFG, left cuneus, and left parahippocampal gyrus than DSU and CTL (see Figure 7 and Table 6). Moreover, PSU and DSU showed lower bilateral thalamus, bilateral middle temporal gyrus, and bilateral cerebellum activation than CTL during the breathing load condition.

**Table 6:** **fMRI results for the group by interoception condition interaction**.

**Volume (μL)**	**No. of voxels in cluster**	***x***	***y***	***z***	**L/R**	**BA**	**Region**
**PSU < OTHER 2 GROUPS (DSU AND CTL) DURING BREATHING LOAD**							
3392	53	22	19	−6	R	11/47	Lentiform nucleus, putamen, inferior frontal gyrus^†^
1856	29	−5	5	−9	L/R	25	Anterior cingulate, subcallosal gyrus^†^
1728	27	1	−62	−16	R	–	Declive^†^
1216	19	−57	−6	0	L	22	Superior/middle temporal gyrus
896	14	−6	−83	37	L	19	Cuneus^†^
704	11	−5	−12	−22	L	–	Parahippocampal gyrus
640	10	24	24	47	R	8	Middle frontal gyrus
576	9	−36	−27	−3	L	–	Lentiform nucleus
576	9	−59	−15	13	L	42	Transverse temporal gyrus
512	8	−10	−92	20	L	18	Cuneus^†^
**CTL > OTHER 2 GROUPS (PSU AND DSU) DURING BREATHING LOAD**							
704	11	−35	−60	−33	L	–	Cerebellar tonsil^†^
640	10	64	−29	−2	R	21	Middle temporal gyrus
512	8	20	−75	−25	R	–	Uvula
512	8	−64	−17	−10	L	21	Middle temporal gyrus^†^
512	8	3	−5	16	L/R	–	Thalamus^†^

L, left hemisphere; R, right hemisphere; BA, Brodmann Area; PSU, Problem Stimulant Users; DSU, Desisted Stimulant Users; CTL, Healthy Comparison Subjects; Coordinates reflect center of mass for each cluster. All clusters emerged as greater than F_(4, 352)_ = 2.96, p = 0.02 corrected voxelwise for multiple comparisons (minimum significant cluster = 8 voxels). Brain activation in all regions above did not correlate with (1) log-transformed lifetime marijuana uses within PSU and DSU; (2) log-transformed interim stimulant uses within PSU; (3) log-transformed BDI-II scores within PSU; (4) BIS-11 Attention scores within PSU.

† Regions that remained significant at F_(4, 352)_ = 3.37, p = 0.01 corrected.

## Discussion

The present study examined how young adults with varying levels of stimulant use differed on neural, behavioral and self-report indices of decision making while experiencing an aversive interoceptive stimulus. Young adults transitioning to PSU show attenuated frontal activations in response to aversive interoceptive stimuli (right IFG/MFG), rewarding outcomes (left SFG), and ambiguous outcomes (right IFG), consistent with hypotheses. In contrast to predictions, however, PSU exhibited heightened frontal activation to punishing outcomes (bilateral IFG), which may be due to the fact that PSU do not register ambiguous feedback as salient via IFG as healthy individuals do (Hampshire et al., 2010). Moreover, it is possible that PSU need to recruit greater IFG to override or ignore aversive non-interoceptive feedback (punishment) but are unable to do so within the context of aversive interoceptive feedback (breathing load) due to an altered homeostatic system (Paulus et al., 2009; Paulus and Stewart, 2014), characterized by reduced thalamic and ACC function. In summary, PSU exhibit impaired somatosensory input via the thalamus and under-recruitment of neural resources to motivate remediation of aversive perturbations via the ACC. In other words, problem users do not exert as many neural resources to process aversive body states and may not integrate these aversive states with ongoing controlled processing, suggesting a disconnect between how “feeling bad” affects a change in “acting.”

In this investigation, five specific hypotheses were examined. First, it was predicted that if attenuated brain activation during decision making indexed current stimulant abuse/dependence, then PSU would exhibit lower neural activation than DSU and CTL while making choices during reward, uncertainty, and/or punishment feedback. PSU exhibited lower right IFG activation than DSU and CTL during uncertain outcomes as hypothesized, but in contrast showed greater bilateral IFG activation than both groups in response to punishing outcomes. Given that healthy individuals demonstrate heightened IFG activation in response to uncertainty (Paulus et al., 2002a; Huettel et al., 2005; Volz et al., 2005; Krain et al., 2006), reduced IFG responses suggest that neural valuation of stimuli is aberrant in PSU. With respect to reward processing, CTL exhibited a pattern of greater left SFG responses to reward than punishment, replicating recent research in healthy individuals (Linke et al., 2010). In contrast, PSU and DSU did not exhibit modulation in this region as a function of valenced feedback. Given that our task as a whole focused on aversive interoceptive manipulation superimposed on rewarding feedback, additional research is needed to determine the role of SFG in stimulant use in various contexts involving reward.

Our second prediction was that PSU would exhibit lower neural activation than DSU and CTL during an aversive interoceptive stimulus. This hypothesis was partially supported, wherein PSU showed lower right IFG/MFG and bilateral subgenual ACC activation than the other two groups. However, both PSU and DSU exhibited lower bilateral thalamic activation than CTL, suggesting that reduced processing of somatosensory inputs (Craig, 2003) may be characteristic of the propensity to try stimulants, rather than a marker of stimulant abuse/dependence. These results suggest that right frontocingulate attenuation, reflecting reduced resources devoted to goal maintenance and action selection in the presence of interoceptive perturbations (May et al., 2013) are indicators of PSU. However, somatosensory reductions via thalamic projections do not appear to be specific to problem use. Third, we hypothesized that lower frontocingulate and insular activation differences between PSU and the other groups would be strongest for the breathing load condition paired with uncertainty (50% error rate). We did not find group differences in insula activation as a function of the aversive interoceptive manipulation with or without uncertainty, suggesting that integration and generation of bodily feeling states associated with insular function may only be impaired in chronic stimulant users.

Fourth, we hypothesized that PSU would report higher subjective unpleasantness ratings of breathing load than DSU and CTL, and overall this prediction was not supported. Although PSU with higher impulsive inattention reported higher ratings of breathing load unpleasantness, this finding was examined *post-hoc* and did not survive multiple comparison thresholds of significance. It could be that in early stages of PSU, individual differences in personality characteristics may play a role in potential aversive interoceptive dysfunction, with a disjointed relationship between subjective feeling states (heightened reported unpleasantness) and neural registration of these feeling states (attenuated insula during unpleasant stimuli). Our findings hint at this possibility, but lack power to robustly support it. Future research is warranted to determine whether larger samples of recent problem users as well as chronic stimulant users show this disjunction moderated by impulsivity.

Our fifth and final prediction was that PSU would employ greater use of a win-stay behavioral strategy than DSU and CTL, a hypothesis that was not supported. *Post-hoc* exploratory analysis suggested a link between greater frequency of stimulant use in the past 3 years and lower win-stay patterns of responding to feedback in the PSU group, a pattern also evident in a recent study of occasional stimulant users (Paulus et al., 2008). However, this correlation did not survive correction for multiple comparisons, limiting further interpretation. Results within our sample indicate that young adults transitioning to problem use do not show similar behavioral impairments as chronic stimulant dependent patients. Perhaps behavioral inflexibility is a result of chronic stimulant use.

Despite several strengths of this study, including use of a novel paradigm and recruitment of young adults at different stages of stimulant use, this investigation possesses several limitations. First, although we had aimed to examine the interaction between decision making and interoception, the two-way error rate by interoception interaction and the three-way group by error rate by interoception condition interaction did not produce significant findings. These null results may be due to an underpowered sample and/or not enough trials within each error rate and interoceptive condition to examine differences. Additional research is warranted to determine the influence of aversive interoceptive stimuli on decision making within the context of different types of valenced feedback in healthy individuals as well as substance users. Second, prior work demonstrates gender differences in neural responses to stress as a function of stimulant dependence (Duncan et al., 2007; Potenza et al., 2012). However, given the relatively small sample sizes for each of our three groups, we are underpowered to reliably examine the role of gender in the present paradigm. Third, although neural indices of interoception and decision making may be differentially altered as a function of the type of stimulant drug used, the modest sample size of groups in the present study did not allow us to examine differences in amphetamine vs. cocaine problem use. Fourth, results indicated that CO_2_ was altered by the aversive interoceptive manipulation across subjects but did not differ as a function of group membership. However, only 2/3 of subjects had usable CO_2_ recordings, which limits the conclusions that can be drawn from this analysis. Future investigations utilizing a large sample of healthy individuals might include CO_2_ levels as a regressor in the fMRI deconvolution analysis to determine its influence on the overall BOLD signal. However, usable CO_2_ data did not differ as a function of group, suggesting that group differences as a function of interoceptive condition cannot be reduced to differences in carbon dioxide levels during breathing load.

This investigation employed a novel task to examine neural and behavioral indicators of decision making and interoception in recent PSU, demonstrating that altered frontocingulate function characterizes young adults transitioning to stimulant dependence. Additional studies are needed to clarify the interoceptive contexts wherein recent and chronic stimulant users exhibit decision making dysfunction.

## Author contributions

Jennifer L. Stewart assisted in data collection, analyzed final fMRI, behavioral, and self-report data, and wrote original draft of manuscript. Jason M. Parnass processed fMRI data up to final analysis, created figures, assisted in data presentation/interpretation, and edited draft of manuscript. April C. May assisted in data collection, created figures, assisted in data presentation/interpretation, and edited draft of manuscript. Paul W. Davenport designed aversive interoceptive manipulation, assisted in data interpretation, and edited draft of manuscript. Martin P. Paulus designed the two-choice prediction task as well as the entire grant-funded project, assisted in data interpretation, and edited draft of manuscript.

### Conflict of interest statement

The authors declare that the research was conducted in the absence of any commercial or financial relationships that could be construed as a potential conflict of interest.

## Footnotes

^1^fMRI data were also analyzed at the standard *p* = 0.01 cutoff using the following LME degrees of freedom and F values: (1) Group main effect: *F* (2, 44) = 5.12; (2) Error rate and interoception condition main effects: *F* (2, 352) = 4.66; (3) Group by error rate, group by condition, and error rate by interoception condition interactions: *F* (4, 352) = 3.37; and (4) Group by error rate by interoception condition interaction: *F*(8, 352) = 2.56. Voxelwise extraction was corrected for multiple comparisons at *p* = 0.01 (minimum cluster size = 7 contiguous voxels).

^2^No findings emerged for the group main effect, error rate by condition interaction, or the group by error rate by condition interaction at *p* = 0.01. For the remaining main effects and interactions presented in Tables 3–6, footnotes under each table highlight which brain regions remained significant at the *p* = 0.01 threshold.

## References

[B1] American Psychiatric Association. (2000). Diagnostic Criteria from DSM-IV-TR. Washington, DC: American Psychiatric Association; xii

[B2] Bechara A. (2005). Decision making, impulse control and loss of willpower to resist drugs: a neurocognitive perspective. Nat. Neurosci. 8, 1458–1463 10.1038/nn1584 16251988

[B3] Beck A. T. Steer R. A. Brown G. K. (1996). Manual for Beck Depression Inventory-II. San Antonio, TX: Psychological Corporation

[B4] Bolla K. I. Eldreth D. A. London E. D. Kiehl K. A. Mouratidis M. Contoreggi C. (2003). Orbitofrontal cortex dysfunction in abstinent cocaine abusers performing a decision-making task. Neuroimage 19, 1085–1094 10.1016/S1053-8119(03)00113-7 12880834 PMC2767245

[B5] Boynton G. M. Engel S. A. Glover G. H. Heeger D. J. (1996). Linear systems analysis of functional magnetic resonance imaging in human V1. J. Neurosci. 16, 4207–4221 8753882 10.1523/JNEUROSCI.16-13-04207.1996 PMC6579007

[B6] Brewer J. A. Bowen S. Smith J. T. Marlatt G. A. Potenza M. N. (2010). Mindfulness-based treatments for co-occurring depression and substance use disorders: what can we learn from the brain. Addiction 105, 1698–1706 10.1111/j.1360-0443.2009.02890.x 20331548 PMC2905496

[B7] Bucholz K. K. Cadoret R. Cloninger C. R. Dinwiddie S. H. Hesselbrock V. M. Nurnberger J. I. Jr. (1994). A new, semi-structured psychiatric interview for use in genetic linkage studies: a report on the reliability of the SSAGA. J. Stud. Alcohol 55, 149–158 8189735 10.15288/jsa.1994.55.149

[B8] Chan P. Y. Davenport P. W. (2008). Respiratory-related evoked potential measures of respiratory sensory gating. J. Appl. Physiol. 105, 1106–1113 10.1152/japplphysiol.90722.2008 18719232 PMC4347743

[B9] Clark V. P. Beatty G. K. Anderson R. E. Kodituwakku P. Phillips J. P. Lane T. D. (2012). Reduced fMRI activity predicts relapse in patients recovering from stimulant dependence. Hum. Brain Mapp. [Epub ahead of print]. 10.1002/hbm.22184 23015512 PMC4470394

[B10] Connolly C. G. Foxe J. J. Nierenberg J. Shpaner M. Garavan H. (2012). The neurobiology of cognitive control in successful cocaine abstinence. Drug Alcohol Depend. 121, 45–53 10.1016/j.drugalcdep.2011.08.007 21885214 PMC3262906

[B11] Cox R. W. (1996). AFNI: software for analysis and visualization of functional magnetic resonance neuroimages. Comput. Biomed. Res. 29, 162–173 10.1006/cbmr.1996.0014 8812068

[B12] Craig A. D. (2003). Interoception: the sense of the physiological condition of the body. Curr. Opin. Neurobiol. 13, 500–505 10.1016/S0959-4388(03)00090-4 12965300

[B13] Critchley H. D. (2004). The human cortex responds to an interoceptive challenge. Proc. Natl. Acad. Sci. U.S.A. 101, 6333–6334 10.1073/pnas.0401510101 15096592 PMC404044

[B14] Critchley H. D. Wiens S. Rotshtein P. Ohman A. Dolan R. J. (2004). Neural systems supporting interoceptive awareness. Nat. Neurosci. 7, 189–195 10.1038/nn1176 14730305

[B15] Damasio A. R. (1996). The somatic marker hypothesis and the possible functions of the prefrontal cortex. Philos. Trans. R. Soc. Lond. B Biol. Sci. 351, 1413–1420 10.1098/rstb.1996.0125 8941953

[B16] Davenport P. W. Vovk A. (2008). Cortical and subcortical central neural pathways in respiratory sensations. Respir. Physiol. Neurobiol. 167, 72–86 10.1016/j.resp.2008.10.001 18977463

[B17] Duncan E. Boshoven W. Harenski K. Fiallos A. Tracy H. Jovanovic T. (2007). An fMRI study of the interaction of stress and cocaine cues on cocaine craving in cocaine-dependent men. Am. J. Addict. 16, 174–182 10.1080/10550490701375285 17612820

[B18] Eddy W. F. Fitzgerald M. Noll D. C. (1996). Improved image registration by using Fourier interpolation. Magn. Reson. Med. 36, 923–931 10.1002/mrm.1910360615 8946358

[B19] Friston K. J. Frith C. D. Turner R. Frackowiak R. S. (1995). Characterizing evoked hemodynamics with fMRI. Neuroimage 2, 157–165 10.1006/nimg.1995.1018 9343598

[B20] Goldstein R. Z. Alia-Klein N. Tomasi D. Zhang L. Cottone L. A. Maloney T. (2007). Is decreased prefrontal cortical sensitivity to monetary reward associated with impaired motivation and self-control in cocaine addiction. Am. J. Psychiatry 164, 43–51 10.1176/appi.ajp.164.1.43 17202543 PMC2435056

[B21] Hampshire A. Chamberlain S. R. Monti M. M. Duncan J. Owen A. M. (2010). The role of the right inferior frontal gyrus: inhibition and attentional control. Neuroimage 50, 1313–1319 10.1016/j.neuroimage.2009.12.109 20056157 PMC2845804

[B22] Haxby J. V. Petit L. Ungerleider L. G. Courtney S. M. (2000). Distinguishing the functional roles of multiple regions in distributed neural systems for visual working memory. Neuroimage 11, 145–156 10.1006/nimg.1999.0527 10679186

[B23] Hoffman W. F. Schwartz D. L. Huckans M. S. McFarland B. H. Meiri G. Stevens A. A. (2008). Cortical activation during delay discounting in abstinent methamphetamine dependent individuals. Psychopharmacology (Berl.) 201, 183–193 10.1007/s00213-008-1261-1 18685833 PMC2835463

[B24] Huettel S. A. Song A. W. McCarthy G. (2005). Decisions under uncertainty: probabilistic context influences activation of prefrontal and parietal cortices. J. Neurosci. 25, 3304–3311 10.1523/JNEUROSCI.5070-04.2005 15800185 PMC6724903

[B25] Kaufman J. N. Ross T. J. Stein E. A. Garavan H. (2003). Cingulate hypoactivity in cocaine users during a GO-NOGO task as revealed by event-related functional magnetic resonance imaging. J. Neurosci. 23, 7839–7843 12944513 10.1523/JNEUROSCI.23-21-07839.2003 PMC6740597

[B26] Krain A. L. Hefton S. Pine D. S. Ernst M. Castellanos F. X. Klein R. G. (2006). An fMRI examination of developmental differences in the neural correlates of uncertainty and decision-making. J. Child Psychol. Psychiatry 47, 1023–1030 10.1111/j.1469-7610.2006.01677.x 17073981

[B27] Kubler A. Murphy K. Garavan H. (2005). Cocaine dependence and attention switching within and between verbal and visuospatial working memory. Eur. J. Neurosci. 21, 1984–1992 10.1111/j.1460-9568.2005.04027.x 15869491

[B28] Leland D. S. Arce E. Feinstein J. S. Paulus M. P. (2006). Young adult stimulant users' increased striatal activation during uncertainty is related to impulsivity. Neuroimage 33, 725–731 10.1016/j.neuroimage.2006.07.011 16959497 PMC1668709

[B29] Linke J. Kirsch P. King A. V. Gass A. Hennerici M. G. Bongers A. (2010). Motivational orientation modulates the neural response to reward. Neuroimage 49, 2618–2625 10.1016/j.neuroimage.2009.09.013 19770058

[B30] May A. C. Stewart J. L. Migliorini R. Tapert S. F. Paulus M. P. (2013). Methamphetamine dependent individuals show attenuated brain response to pleasant interoceptive stimuli. Drug Alcohol Depend. 131, 238–246 10.1016/j.drugalcdep.2013.05.029 23806873 PMC3760794

[B31] Monterosso J. R. Ainslie G. Xu J. Cordova X. Domier C. P. London E. D. (2007). Frontoparietal cortical activity of methamphetamine-dependent and comparison subjects performing a delay discounting task. Hum. Brain Mapp. 28, 383–393 10.1002/hbm.20281 16944492 PMC6871407

[B32] Naqvi N. H. Bechara A. (2010). The insula and drug addiction: an interoceptive view of pleasure, urges, and decision-making. Brain Struct. Funct. 214, 435–450 10.1007/s00429-010-0268-7 20512364 PMC3698865

[B33] Nestor L. J. Ghahremani D. G. Monterosso J. London E. D. (2011). Prefrontal hypoactivation during cognitive control in early abstinent methamphetamine-dependent subjects. Psychiatry Res. 194, 287–295 10.1016/j.pscychresns.2011.04.010 22047731 PMC3225642

[B34] Oldfield R. C. (1971). The assessment and analysis of handedness: the Edinburgh inventory. Neuropsychologia 9, 97–113 10.1016/0028-3932(71)90067-4 5146491

[B35] Patton J. H. Stanford M. S. Barratt E. S. (1995). Factor structure of the Barratt impulsiveness scale. J. Clin. Psychol. 51, 768–774 10.1002/1097-4679(199511)51:6<768::AID-ICLD2270510607>3.0co;2-1 8778124

[B36] Paulus M. P. Flagan T. Simmons A. N. Gillis K. Kotturi S. Thom N. (2012). Subjecting elite athletes to inspiratory breathing load reveals behavioral and neural signatures of optimal performers in extreme environments. PLoS ONE 7:e29394 10.1371/journal.pone.0029394 22276111 PMC3261851

[B37] Paulus M. P. Hozack N. Frank L. Brown G. G. (2002a). Error rate and outcome predictability affect neural activation in prefrontal cortex and anterior cingulate during decision-making. Neuroimage 15, 836–846 10.1006/nimg.2001.1031 11906224

[B38] Paulus M. P. Hozack N. E. Zauscher B. E. Frank L. Brown G. G. Braff D. L. (2002b). Behavioral and functional neuroimaging evidence for prefrontal dysfunction in methamphetamine-dependent subjects. Neuropsychopharmacology 26, 53–63 10.1016/S0893-133X(01)00334-7 11751032

[B39] Paulus M. P. Hozack N. Frank L. Brown G. G. Schuckit M. A. (2003). Decision making by methamphetamine-dependent subjects is associated with error-rate-independent decrease in prefrontal and parietal activation. Biol. Psychiatry 53, 65–74 10.1016/S0006-3223(02)01442-7 12513946

[B40] Paulus M. P. Lovero K. L. Wittmann M. Leland D. S. (2008). Reduced behavioral and neural activation in stimulant users to different error rates during decision making. Biol. Psychiatry 63, 1054–1060 10.1016/j.biopsych.2007.09.007 17949691 PMC4286246

[B41] Paulus M. P. Stewart J. L. (2014). Interoception and drug addiction. Neuropharmacology 76, 342–350 10.1016/j.neuropharm.2013.07.002 PMC3858461 23855999

[B42] Paulus M. P. Tapert S. F. Schuckit M. A. (2005). Neural activation patterns of methamphetamine-dependent subjects during decision making predict relapse. Arch. Gen. Psychiatry 62, 761–768 10.1001/archpsyc.62.7.761 15997017

[B43] Paulus M. P. Tapert S. F. Schulteis G. (2009). The role of interoception and alliesthesia in addiction. Pharmacol. Biochem. Behav. 94, 1–7 10.1016/j.pbb.2009.08.005 19698739 PMC2753707

[B44] Pinheiro J. Bates D. DebRoy S. Sarkar D. R Core Team (2013). nlme: linear and nonlinear mixed effects models. R package Version 3.1-111. Avaliable online at: http://cran.r-project.org/web/packages/nlme/citation.html

[B45] Pollatos O. Schandry R. Auer D. P. Kaufmann C. (2007). Brain structures mediating cardiovascular arousal and interoceptive awareness. Brain Res. 1141, 178–187 10.1016/j.brainres.2007.01.026 17296169

[B46] Porges S. W. (1993). Body Perception Questionnaire: Laboratory of Developmental Assessment. College Park, MD: University of Maryland

[B47] Potenza M. N. Hong K. I. Lacadie C. M. Fulbright R. K. Tuit K. L. Sinha R. (2012). Neural correlates of stress-induced and cue-induced drug craving: influences of sex and cocaine dependence. Am. J. Psychiatry 169, 406–414 10.1176/appi.ajp.2011.11020289 22294257 PMC3690485

[B48] R-Development-Core-Team. (2008). R: A Language and Environment for Statistical Computing. Vienna: R Foundation for Statistical Computing

[B49] Reske M. Delis D. C. Paulus M. P. (2011). Evidence for subtle verbal fluency deficits in occasional stimulant users: quick to play loose with verbal rules. J. Psychiatr. Res. 45, 361–368 10.1016/j.jpsychires.2010.07.005 20673916 PMC3424267

[B50] Salo R. Ursu S. Buonocore M. H. Leamon M. H. Carter C. (2009). Impaired prefrontal cortical function and disrupted adaptive cognitive control in methamphetamine abusers: a functional magnetic resonance imaging study. Biol. Psychiatry 65, 706–709 10.1016/j.biopsych.2008.11.026 19136097 PMC2678684

[B51] Sinha R. (2007). The role of stress in addiction relapse. Curr. Psychiatry Rep. 9, 388–395 10.1007/s11920-007-0050-6 17915078

[B52] Sinha R. Fox H. Hong K. I. Sofuoglu M. Morgan P. T. Bergquist K. T. (2007). Sex steroid hormones, stress response, and drug craving in cocaine-dependent women: implications for relapse susceptibility. Exp. Clin. Psychopharmacol. 15, 445–452 10.1037/1064-1297.15.5.445 17924778

[B53] Sinha R. Garcia M. Paliwal P. Kreek M. J. Rounsaville B. J. (2006). Stress-induced cocaine craving and hypothalamic-pituitary-adrenal responses are predictive of cocaine relapse outcomes. Arch. Gen. Psychiatry 63, 324–331 10.1001/archpsyc.63.3.324 16520439

[B54] Sinha R. Lacadie C. Skudlarski P. Fulbright R. K. Rounsaville B. J. Kosten T. R. (2005). Neural activity associated with stress-induced cocaine craving: a functional magnetic resonance imaging study. Psychopharmacology (Berl.) 183, 171–180 10.1007/s00213-005-0147-8 16163517

[B55] Skudlarski P. Constable R. T. Gore J. C. (1999). ROC analysis of statistical methods used in functional MRI: individual subjects. Neuroimage 9, 311–329 10.1006/nimg.1999.0402 10075901

[B56] Spielberger C. D. Gorsuch R. L. Lushene R. E. Vagg P. R. (1983). State-trait anxiety inventory (STAI). BiB 2010, 180

[B57] SPSS. (2009). PASW statistics for windows, version 18.0. Chicago, IL: SPSS Inc

[B58] Stewart J. L. Flagan T. M. May A. C. Reske M. Simmons A. N. Paulus M. P. (2013). Young adults at risk for stimulant dependence show reward dysfunction during reinforcement-based decision making. Biol. Psychiatry 73, 235–241 10.1016/j.biopsych.2012.08.018 23021534 PMC3674030

[B59] Tiffany S. T. Singleton E. Haertzen C. A. Henningfield J. E. (1993). The development of a cocaine craving questionnaire. Drug Alcohol Depend. 34, 19–28 10.1016/0376-8716(93)90042-O 8174499

[B60] Tomasi D. Goldstein R. Z. Telang F. Maloney T. Alia-Klein N. Caparelli E. C. (2007a). Widespread disruption in brain activation patterns to a working memory task during cocaine abstinence. Brain Res. 1171, 83–92 10.1016/j.brainres.2007.06.102 17765877 PMC2048813

[B61] Tomasi D. Goldstein R. Z. Telang F. Maloney T. Alia-Klein N. Caparelli E. C. (2007b). Thalamo-cortical dysfunction in cocaine abusers: implications in attention and perception. Psychiatry Res. 155, 189–201 10.1016/j.pscychresns.2007.03.002 17582746 PMC2265105

[B62] Verdejo-Garcia A. Clark L. Dunn B. D. (2012a). The role of interoception in addiction: a critical review. Neurosci. Biobehav. Rev. 36, 1857–1869 10.1016/j.neubiorev.2012.05.007 22659642

[B63] Verdejo-Garcia A. Contreras-Rodriguez O. Fonseca F. Cuenca A. Soriano-Mas C. Rodriguez J. (2012b). Functional alteration in frontolimbic systems relevant to moral judgment in cocaine-dependent subjects. Addict. Biol. 32, 494–501 10.1111/j.1369-1600.2012.00472.x 22784032

[B64] Verdejo-Garcia A. Lawrence A. J. Clark L. (2008). Impulsivity as a vulnerability marker for substance-use disorders: review of findings from high-risk research, problem gamblers and genetic association studies. Neurosci. Biobehav. Rev. 32, 777–810 10.1016/j.neubiorev.2007.11.003 18295884

[B65] Volz K. G. Schubotz R. I. von Cramon D. Y. (2005). Variants of uncertainty in decision-making and their neural correlates. Brain Res. Bull. 67, 403–412 10.1016/j.brainresbull.2005.06.011 16216687

[B66] Zaki J. Davis J. I. Ochsner K. N. (2012). Overlapping activity in anterior insula during interoception and emotional experience. Neuroimage 62, 493–499 10.1016/j.neuroimage.2012.05.012 22587900 PMC6558972

[B67] Zuckerman M. (2007). The sensation seeking scale V (SSS-V): Still reliable and valid. Pers. Individ. Dif. 43, 1303–1305 10.1016/j.paid.2007.03.021

